# Design of a Modular Protein-Based MRI Contrast Agent for Targeted Application

**DOI:** 10.1371/journal.pone.0065346

**Published:** 2013-06-06

**Authors:** Daniel Grum, Stefan Franke, Oliver Kraff, Dominik Heider, Alexander Schramm, Daniel Hoffmann, Peter Bayer

**Affiliations:** 1 Research Group Structural and Medicinal Biochemistry, Centre for Medical Biotechnology, University of Duisburg-Essen, Essen, Germany; 2 Research Group Bioinformatics, Centre for Medical Biotechnology, University of Duisburg-Essen, Essen, Germany; 3 Erwin L. Hahn Institute for Magnetic Resonance Imaging, University of Duisburg-Essen, Essen, Germany; 4 Department of Pediatric Hematology, University Children’s Hospital, Essen, Germany; Stanford, United States of America

## Abstract

Magnetic resonance imaging (MRI) offers a non-radioactive alternative for the non-invasive detection of tumours. Low molecular weight MRI contrast agents currently in clinical use suffer either from a lack of specificity for tumour tissue or from low relaxivity and thus low contrast amplification. In this study, we present the newly designed two domain fusion protein Zarvin, which is able to bind to therapeutic IgG antibodies suitable for targeting, while facilitating contrast enhancement through high affinity binding sites for Gd^3+^. We show that the Zarvin fold is stable under serum conditions, specifically targets a cancer cell-line when bound to the Cetuximab IgG, and allows for imaging with high relaxivity, a property that would be advantageous for the detection of small tumours and metastases at 1.5 or 3 T.

## Introduction

Early diagnosis of cancer and metastatic disease is highly correlated to therapeutic success in the majority of solid malignancies. The state of the art for detection and localization of small tumours and metastases are the multimodality techniques PET/CT and SPECT/CT [1]. However, these techniques rely on X-rays and contrast agents based on radio isotopes. In order to avoid exposure of patients to ionizing radiation, alternative methods have been developed like magnetic resonance imaging (MRI) with high-relaxivity contrast agents such as small molecular weight and protein binding blood pool agents, nanoparticles, dendrimers, liposomes and proteins (e.g. references [2], [3], [4], [5]). Most of these contrast agents contain the lanthanide ion Gd^3+^, which produces positive contrast in T_1_ weighted imaging. Concerning the design of high-relaxivity contrast agents for MRI, two main approaches have emerged over time [6]. While one strategy aims to link multiple small molecular weight agents, another attempts to optimize molecular parameters influencing relaxivity of an agent, especially the molecular weight. Furthermore, combining enhanced relaxivity with a targeting approach to achieve high local concentrations of the contrast agent was successfully demonstrated in animal experiments [7], [8]. Our approach was to design a high-relaxivity targeted contrast agent by increasing the molecular weight of the probe. Here, we report the design of a protein-based T_1_ contrast agent named Zarvin. Zarvin comprises several parts. The first part is the Z domain of *Staphylococcus aureus* [9] protein A for non-covalent binding to antibodies. The Z domain is linked to a S55D/E59D mutant of the Ca^2+^ binding rat alpha-Parvalbumin [10] for binding of Gd^3+^ and for achieving high relaxivity. We demonstrate *in-vitro*, including cell targeting assays, that Zarvin allows for various targeting applications in combination with commercially available antibodies and without the need of antibody modification. Our experiments point to optimization towards *in-vivo* application.

## Materials and Methods

### Modelling

All structural templates were taken from the Protein Data Bank (PDB) [20]. Three dimensional models of Zarvin were generated with the MODELLER 9.1 [21] software, using as templates the Z domain (PDB entry 1q2n [22]) and the calcium bound structure of Parvalbumin (PDB entry 1s3p [10]). The C-terminus of the Z domain was linked to the N-terminus of Parvalbumin with a decaglycine. In total, we modelled 50 structures.

### Clustering and Secondary Structure Prediction

We used a cluster algorithm [23] to group the models according to a RMSD cut-off of 1.7 Å. For one structure in each cluster, we performed a molecular dynamics (MD) simulation. DSSP [24] was used to analyse the secondary structure.

### Molecular Dynamics Simulations

MD simulations were performed in the NPT ensemble at 300K and atmospheric pressure using GROMACS 4.0 [25] with the GROMOS 43a1 force field [26] and SPC/E water. Temperature and pressure was stabilized at 300 K by Nose-Hoover thermostat and 1 atm by Parinello-Rahman barostat, respectively. Periodic boundary conditions with triclinic boxes were applied with a minimum of 1.0 nm distance between protein and faces of box. Residues were assumed to be protonated according to their normal states at pH 7. Na^+^ and Cl^−^ ions were added to neutralize the system at an ionic strength of 0.15 mol/l. The Particle Mesh Ewald method was used to compute electrostatic interactions boundary conditions. Bonds involving hydrogen atoms were constrained using the SHAKE algorithm [27], allowing for a time step of 2 fs. Structures were energy minimized and equilibrated by molecular dynamics for 1 ns. Snapshots of the trajectories were saved every 100 ps. In total, we simulated the system for 200 ns. The last 5 ns of all trajectories were analyzed with the toolbox provided by GROMACS, especially g_rms for root-mean-square-deviations (RMSDs) and g_rmsf for root-mean-square-fluctuations (RMSFs). Due to the physical-chemical similarity of Ca^2+^ and Gd^3+^, we used the default Ca^2+^ parameter.

### Distance Analysis and Water Coordination Analysis

To analyse the dynamic behaviour of the system, we calculated the distance between the centre of mass (COM) of both domains using the g_dist program available in the Gromacs package. g_dist was also used to calculate the number of water molecules that are present in the first coordination sphere of Ca^2+^. We determined the number of water molecules in a distance of 3.4 Å to the calcium ion, the maximum distance between the calcium ion and a water molecule in the first coordination sphere according to Plazinski et al. [28]. To check for intramolecular interactions, we calculated the minimum distance between the atoms of both domains using the g_mindist program of the Gromacs package.

### Cloning and Expression

The gene encoding the two-domain protein Zarvin was commercially synthesised and subcloned in a derivative of a pET41b expression vector containing GST and a PreScission cleavage site, both located N-terminal of the multiple cloning site (Geneart, Regensburg, Germany). The cloning sites used were ApaI and BamHI. The single Z- and Parvalbumin-domains were subcloned in the same vector by first amplifying the respective genes from the full length construct via PCR. The primers used for Z domain subcloning were 5′ CACACAGGGCCCGTGGATAACAAATTTAACAAAGAACAGC-3′ for forward ApaI cloning and 5′ GGTTGGGGATCCATTATTTCGGCGCCTGCGCATCGT 3′ for reverse BamHI cloning. The primers used for rat S55D/E59D-alpha-Parvalbumin subcloning were 5′ CACACAGGGCCCAGCATGACCGATCTGCTGAGCGC 3′ for forward ApaI cloning and 5′ GGTTGGGGATCCATTAGCTTTCCGCCACCAGGG 3′ for reverse BamHI cloning. Mutation of Zarvin yielding a D72C mutant was performed using a Quik-Change® kit (Stratagene). The respective primers used for mutation were forward 5′ GGCAGCATGACCTGTCTGCTGAGC 3′ and reverse 5′ GCTCAGCAGACAGGTCATGCTGCC 3′.

Recombinant Zarvin was expressed as GST fusion protein. Expression was performed in E. coli BL21(DE3)T1r cells. For that, after cells were grown up to OD 0.6 in 2× YT medium at 37°C protein expression was induced with 0.2 mM IPTG. Expression was performed for another 5 h at 29°C. Bacteria pellets were suspended in 20 mM Tris, 50 mM NaCl, pH 7.4 and cells were cracked via lysozyme treatment for 1 h at 4°C following sonication at room temperature. The supernatant of subsequent ultracentrifugation was bound to a 20 ml GSTrap column (GE Healthcare) using a BioRad FPLC at 4°C. Bound protein was washed with three buffers to remove unwanted components. First, a high salt buffer consisting of 20 mM Tris, 500 mM NaCl, pH 7.4 was used to remove unspecifically bound proteins from the column. A buffer consisting of 20 mM Tris, 150 mM NaCl, 70 mM EDTA, pH 7.4 was used to remove potentially bound Ca^2+^ ions from the metal ion binding sites of the Parvalbumin domain. Another buffer consisting of 20 mM Tris, 150 mM NaCl, 0.2 mM ATP, pH 7.4 was used to remove potentially bound bacterial chaperones. Elution was performed using 20 mM Tris, 150 mM NaCl, 20 mM glutathione, pH 7.4. Eluted fusion protein was concentrated and glutathione was removed via dialysis using a 30 kDa MWCO centricon (Millipore) by repeated centrifugation and refilling of the centricon. Afterwards, cleavage with PreScission protease was performed at 4°C over night. Zarvin was purified by first removing GST via a 20 ml GSTrap column. The flow-through was collected and concentrated again. Gelfiltration using a Superdex 75 26/60 column (GE Healthcare) was performed to remove aggregates and degraded protein. The buffer used for GST removal as well as for gel filtration contained 20 mM Tris, 150 mM NaCl, pH 7.4. Purified protein was aliquoted, shock frozen and stored at −20°C.

The D72C mutant of Zarvin and the separate Parvalbumin domain were purified in the same way, however 1 mM DTT was added to all buffers for purification of Zarvin-D72C. The separate Z domain was also purified as GST fusion protein, only missing the washing step with EDTA. Due to the architecture of the PreScission cleavage site the two amino acids GP remain at the N terminus of the protein.

### MALDI-MS

Proteins were desalted using Supel-Tips C18 (Sigma) and eluted with 50∶50 (v/v) acetonitrile: 0.1% TFA in water. Matrix solution was prepared by dissolving 7.6 mg 2′,5′-dihydroxy acetophenone in ethanol and adding 125 µl of a solution containing 18 mg/ml di-ammonium hydrogen citrate in water. Proteins were spotted on a Ground steel target plate (Bruker Daltonics) using the dried droplet method. For that, 2% TFA in water were mixed with the desalted protein and matrix solution in a ratio of 1∶1∶1 (v/v). The spot was dried at room temperature. Mass analysis was carried out using an Autoflex speed MALDI-TOF (Bruker Daltonics).

### Analytical Gelfiltration

Gelfiltration experiments were performed using a Superdex 75 10/300 GL or a Superdex 75 16/26 column (GE Healthcare) and a BioRad FPLC at 4°C. For calibrating the column Alpha-Amylase (porcine pancreas), Ovalbumin (Sigma), GAPDH from rabbit muscle (Sigma), Chymotrypsinogen A from bovine pancreas and Lysozyme (Fluka) were used. Runs were performed at 0.5 ml/min in 20 mM Tris, 500 mM NaCl, pH 7.4 and 20 mM Tris, 200 mM NaCl, 3 M guanidine hydrochloride, pH 7.4.

### Protein Concentration Determination

Protein concentrations of stock solutions were determined by measuring the absorbance of the solution at 280 nm or 258 nm, respectively, using a NanoDrop ND 1000 (peqlab). Absorbance was measured at least three times and averaged following concentration determination using an extinction coefficient of 1490 M^−1^cm^−1^ for Zarvin as well as for the Z domain and 217315 M^−1^cm^−1^ for Cetuximab. Parvalbumin concentrations were determined by measuring the absorbance at 258 nm and using an extinction coefficient of 1512 M^−1^cm^−1^, which is the sum of the absorbances of eight phenylalanine residues. It was calculated with an absorbance of 189 M^−1^cm^−1^ at 258 nm for one phenylalanine [29]. Exact protein concentrations of samples where CD spectra were recorded from were measured using a Cary 100 UV/Vis spectrophotometer.

### CD Spectroscopy

CD spectroscopy was performed using a JASCO J815 CD spectrometer. For recording of spectra Zarvin was diluted in 20 mM Natrium phosphate buffer, pH 7.4 at a final concentration of approximately 0.15 mg/ml and using a 1 mm cuvette. CD spectra were recorded from 200–260 nm. Further parameters were data pitch: 0.2 nm, scanning mode: continuous, scanning speed: 100 nm/min, response time: 0.5 s, bandwidth: 2 nm. 15 scans were averaged for each spectrum. Spectra were recorded at room temperature. Data processing was performed by separately shifting the blank and the protein spectrum to zero for the wavelengths 250–260 nm and subsequent subtraction of the blank spectrum from the protein spectrum. Afterwards, the mdeg units of the spectrum were transformed to Δε MRW units using a mean residue weight (MRW) of 107.091 Da for Zarvin and the spectroscopically determined concentration of the protein in mg/ml units. These data processing steps were performed using unpublished software (Steve Martin, NIMR London). Secondary structure contents of Zarvin were calculated using the programs CDSSTR, Contin ll and Selcon 3. The complete range of secondary structure contents calculated by the three programs is displayed in table S1. Melting curves of Zarvin and the single domains were performed by diluting the proteins in 20 mM HEPES, 20 mM NaCl, pH 7.4 at a final concentration of 0.09 mg/ml and using a 2 mm cuvette. Recording parameters used were data pitch: 0.1°C, delay time: 180 s, bandwidth: 1 nm, response time: 8 s, temperature slopes: 0.5°C/min for melting and 1°C/min for cooling.

### Labelling of Zarvin

N-terminal labelling of Zarvin as well as labelling of the cysteine residue of Zarvin-D72C was performed using NHS-Ester and maleimide derivatives of Atto dyes respectively (Atto-tec, Siegen, Germany). Labelling was performed according to the Atto-tec protocols for amine and thiol reactive dyes respectively. The chosen labelling strategy was 1 h at room temperature and subsequent separation of the protein-dye complex from unbound dye using two PD-10 columns (GE Healthcare) in a row. Corrected protein concentrations of the protein-dye complexes and labelling efficiencies were determined spectroscopically using a Cary 100 UV/Vis spectrophotometer. If not used immediately, labelled protein was shock frozen and stored at −20°C.

### Fluorescence Anisotropy Titrations

Fluorescence anisotropy measurements were performed using a Cary eclipse fluorescence spectrometer (Agilent Technologies) equipped with a polariser. Approximately 120 nM of Zarvin N-terminally labelled with Atto-465 was titrated with the monoclonal antibody Cetuximab by recording the fluorescence anisotropy signal of Atto-465. The buffer used was 20 mM Tris, 150 mM NaCl, pH 7.4. Measurements were performed using following parameters: 440 nm for excitation of Atto-465 and 525 nm for emission, excitation slit width: 10 nm, emission slit width: 20 nm, PMT voltage: 600 V, temperature: 20°C, G-factor for Atto-465∶1.6848. Data points of the titration were recorded in the kinetic mode using an average time of 7 s and a recording time of at least 10 min for each point.

### Cell Culture and Fluorescence Microscopy

Binding of the complex of Cetuximab and Zarvin-D72C-Atto594 to the cell membrane bound EGF receptor of A431 [19] cells was visualised by fluorescence microscopy using a Leica SP5 confocal microscope. For that, A431 cells were grown to a density of 10^5^–10^6^ cells/cm^2^ on µ-Slide 8 well plates (Ibidi). Afterwards, medium was removed and cells were incubated with a mixture of Cetuximab and Zarvin-D72C-Atto594, Cetuximab alone, Zarvin-D72C-Atto594 alone or buffer without any protein for 30 minutes at room temperature. The used buffer was 25 mM HEPES, 150 mM NaCl, 4 mM KCl, pH 7.4. A431 cells are viable in this buffer for days. Protein concentrations were 7.8 µM Cetuximab and 14 µM Zarvin-D72C-Atto594 in HEPES buffer. Finally, unbound protein was removed by 5 washing steps with HEPES buffer and cells were left in this buffer containing 10% FCS during microscopy. Atto-594 was excited with a 594 nm laser and an intensity of 50%. Emission was detected between 605–750 nm with a PMT voltage between 800 volts. Auto fluorescence of cells was measured by exciting with a 405 nm diode laser and detecting fluorescence between 417–502 nm. The laser intensity used was 70% and the PMT voltage 820 V. Pictures were recorded with a resolution of 8 bit, a size of 1024×1024 pixel, a line average of 8 and a scanning speed of 200 pixel/sec. Z-stacks were recorded with a stack thickness of 0.17 µM, a size of 1024×1024 pixel, a line average of 2 and a scanning speed of 400 pixel/sec. Image processing concerning brightness and contrast adjustments were done using the program Image J.

### Luminescence Measurements and Metal Titrations

Luminescence measurements were performed using a Cary eclipse fluorescence spectrometer. Terbium (III) in complex with Zarvin was excited via energy transfer from a phenylalanine between the EF and CD metal binding sites of the Parvalbumin domain. The excitation wavelength used for this was 258 nm. Luminescence emission was detected at 543 nm (5D4 → 7F5 transition) with a delay time of 0.2 ms, a gate time of 4.5 ms and a total decay time of 200 ms. The excitation and emission slit widths used were 10 nm and 20 nm respectively. The PMT voltage was 800 V. Titrations were carried out at 20°C. Each titration step was measured in kinetic mode with an average time of 7 s over a time scale of 10 minutes.

Affinity determinations of Tb^3+^ to the EF and CD site of the Parvalbumin domain were done in 20 mM Tris, 150 mM NaCl, pH 7.4. Each titration step was pipetted separately and all batches were incubated for three days at room temperature to establish equilibrium between the complexes Zarvin:(Tb^3+^)_2_ and NTA:Tb^3+^. The curve was normalised and inverted prior to fitting with a Hill equation. Using the fitted apparent affinity of NTA:Tb^3+^ and the real binding affinity [15] of this complex of 5.6 × 10^−12^ M, the binding affinity of Zarvin:(Tb^3+^)_2_ was estimated according to equation:
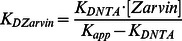
(1)where K_app_ is the fitted apparent K_D_ of NTA. This equation is a transposed form of an equation derived for calculating real binding constants using Kapp and [competitor] from competition titrations [30]:
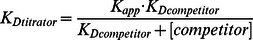
(2)where in our case NTA is the titrator and Zarvin the competitor. Competition titrations where Tb3+ bound to Zarvin was displaced by either Gadolinium (III) or Calcium (II) were conducted in 20 mM Tris, 150 mM NaCl, pH 7.4. The affinities of Gd3+ and for comparison of Ca2+ were measured using a competition assay where 4 µM Zarvin and 10 µM Tb3+ were titrated with Gd3+ or Ca2+ respectively. Data were fitted using a hyperbolic equation for competition titrations [31]:
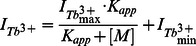
(3)with ITb3+ being the Tb3+ luminescence intensity and [M] the concentration of Gd3+ or Ca2+ respectively.

### Nonlinear Regression

Nonlinear regression for all titrations was conducted using the program Graphpad prism (Graphpad Software).

### Relaxometry

Relaxometric analysis of the complex of Zarvin and two Gd^3+^ ions was carried out at three different field strengths, 1.5 T, 3 T and 7 T, and at room temperature. Imaging was performed at all three field strengths using a whole-body MRI scanner (Magnetom Aera, Skyra and 7 T; Siemens Healthcare Sector, Erlangen, Germany) capable of 45 mT/m (70 mT/m at 7 T) gradient strength and 200 mT/m/s slew rate. The buffer used was 20 mM HEPES, 150 mM NaCl, 4 mM KCl, pH 7.4. Samples were put in a 4 × 6 well TC-Plate (Greiner bio-one) at a volume of 2.9 ml each. Concentrations of 20, 10, 5, 1, 0.5 and 0 µM Zarvin were used each containing a slightly under saturated double amount of Gd^3+^ (39.5, 19.7, 9.9, 1.97, 0.99 and 0 µM). The plate was positioned within the RF coil. A 16-channel receive wrist coil was used at 1.5 T and 3 T (Siemens Healthcare Sector), while at 7 T a 32-channel transmit/receive head coil (Nova Medical, New York, USA) was used. For the determination of T1 relaxation time constants, inversion recovery turbo spin echo (IR-TSE) sequences with the following parameters were used: inversion times (T_I_) between 20 and 2500 ms; repetition time/echo time (T_R_/T_E_) 12500/6.8 ms; field of view (FoV) read 180 mm; FoV phase 100.0%; frequency resolution 384; phase resolution 100%; bandwidth 395 Hz/Px; slice thickness 3 mm, nominal flip angle 180 degrees, turbo-factor 11.

The resolution of the images recorded is calculated from FOV/Matrix in READ x PHASE and the slice thickness, so 0.5×0.5×3 mm^3^ equalling 0.75 mm^3^. The inversion time zeroing the MRI signal (sample appears black) was used for calculating the T_1_ relaxation time by T_1_ = - T_I_(0)/ln(0.5).

The longitudinal relaxation time constant T_1_ was calculated for each individual contrast agent at all measured concentrations and field strengths [32]. T_1_ relaxivity r_1_ in units of s^−1^mM^−1^ further was calculated according to (1/T_1_–1/T_1buffer_)/c(Gd^3+^) and was averaged over the three wells containing 20, 10 and 5 µM Zarvin. The relaxation rate constants (R_1_) were calculated according to the equation R_1_ = 1/T_1_. The relaxivities (r_1_) were calculated using r1 = (1/T_I_(c) - 1/T_I_(0))/c where, T_I_(c) is the relaxation time constant at concentration c; T_I_(0) is the relaxation time constant of the solvent without contrast agent and c is the concentration of a given contrast agent. The T_I_(0) values found for the three Zarvin concentrations were 300, 500, 800 ms for 1.5 T, 500, 800 and 1100 ms for 3 T and 900, 1350 and 1600 ms for 7 T.

The NMRD profile of a 0,48 mM sample of Zarvin:(Gd^3+^)_2_ in 20 mM Tris, 150 mM NaCl, pH 7.4 was measured in cooperation with the company Stelar Srl (Mede (PV), Italy). Longitudinal relaxation curves of the sample were measured at a number of magnetic field strength values in the range 0.01–70 MHz, using:

- Fast Field Cycling NMR Relaxometer produced by Stelar Srl. Standard Pre-Polarized (PP/S) and Non-Polarized.

(NP/S) acquisition sequences have been used with the following parameters:
  Temperature [°C]: 37.
  BRLX Relaxation field [MHz on ^1^H]: 0.01–35.
  BACQ Acquisition field [MHz on ^1^H]: 16.3.
  NUC Nucleus ^1^H.
  SF Spectrometer frequency [MHz]: 16.3.
  MS Number of scans 1.
  PW90 90° pulse width [µs]: 9.6.

- WP80 electromagnet with Inversion Recovery (180°- tau- 90°)n sequence.

Temperature [°C]: 37.
  B0 Magnetic Field [MHz on ^1^H]: 20–70.
  MS Number of scans 2.
  PW90 90° pulse width [µs]: 4.5.
  A set of 32 relaxation interval values (tau) allowed description of the spin-lattice decay curves for each magnetic field strength.
  The temperature was controlled by a Stelar VTC-91 airflow heater, equipped with a copper-constantan thermocouple. The temperature calibration in the probe head was carried out with a Delta OHM digital thermometer, with an absolute accuracy of 0.5°C.

### NMR Analysis

^1^H-^15^N-HSQC spectra of Zarvin, the S55D/E59D rat alpha-Parvalbumin and the Z domain were recorded with protein samples of 0.1 to 0.5 mM (in 20 mM Tris, 150 mM NaCl, pH 7,4; 10% D_2_O: 90% H_2_O) at 300 K using an Ultrashield™ 700 NMR spectrometer equipped with a triple resonance Cryoprobe head (Bruker Biospin, Fällanden). The proteins were labeled with ^15^N by growing the used BL21(DE3)T1r *E. coli* cells in M9 minimal medium supplemented with ^15^N ammonium chloride. 17 mM CaCl_2_ has been added to the Parvalbumin and Zarvin samples. Spectra were processed with TOPSPIN 3.0 (Bruker Biospin, Rheinstetten) and evaluated and analyzed using the software CcpNmr Analysis [33].

## Results and Discussion

**Figure 1:**
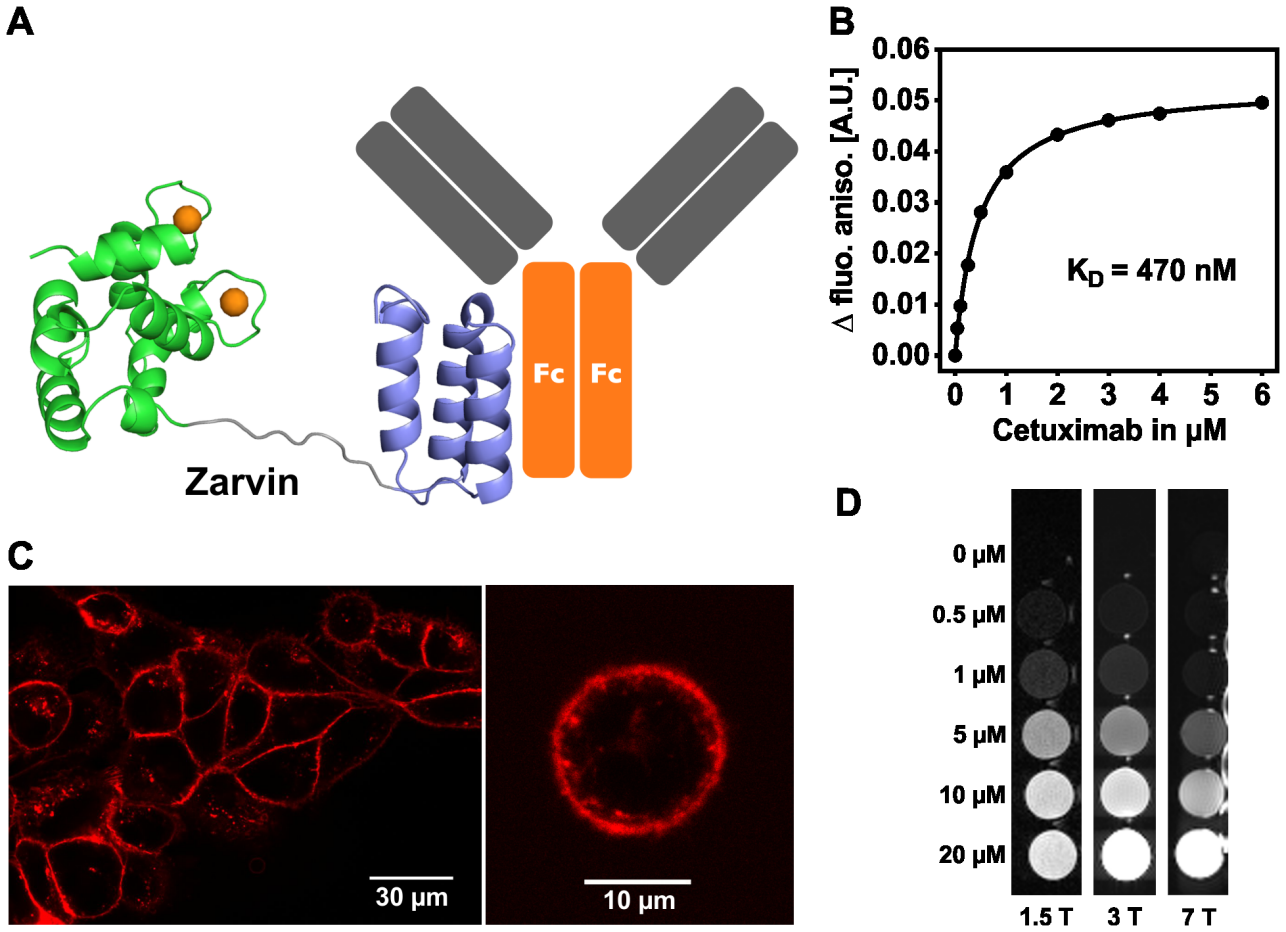
**Binding properties and relaxometric properties of Zarvin.** (A) Cartoon representation of Zarvin bound to the F_c_ part of an IgG antibody. Two calcium ions (spheres) are bound to Parvalbumin (green), which is connected with the Z domain (violet) via a decaglycine linker (grey). (B) Fluorescence anisotropy titration experiment. Increasing amounts of the monoclonal IgG antibody Cetuximab were added to a 100 nM concentration of Zarvin-Atto-465. (C) Confocal microscopic analysis of the complex Cetuximab:Zarvin-D72C-Atto 594 binding to the EGF receptor located in the cell membrane of A431 cells. Left, cell assembly; right, single cell; control experiments (Figure S4) (D) Relaxometric properties of Zarvin:(Gd^3+^)_2_ at three different field strengths employing an inversion recovery TSE experiment. A diluted solution of rising concentrations of Zarvin:(Gd^3+^)_2_ was investigated to find the limiting concentration which still produces a visible contrast towards the buffer control (0 µM). The picture is displayed with an inversion time TI which zeroes the signal of the buffer control (appears black).

First, Zarvin was modelled and studied using in-silico methods. For this purpose three-dimensional models of Zarvin were generated with the MODELLER 9.1 software, using the Z domain (PDB entry 1q2n) and the calcium bound structure of S55D/E59D alpha-Parvalbumin (PDB entry 1s3p) as templates. The C-terminus of the Z domain was linked to the N-terminus of Parvalbumin using a decaglycine peptide (Figure 1A).

To investigate the stability of the system and to estimate the usage as contrast agent molecular dynamics (MD) simulations were performed with the GROMACS 4.0 software. In total, we simulated the system for 100 ns. A detailed description can be found in the supplementary materials. MD simulations showed that while the secondary and tertiary structure of the Z domain and of modified Parvalbumin are conserved, the two domains are free to re-orient independently of each other (Figure S1). Furthermore, the number of water molecules in the first Ca^2+^ coordination sphere was calculated as test for the suitability as contrast agent. The main water coordination number is one, which is comparable with the coordination number of Gd^3+^ in clinically used chelates (Figure S2). In about one third of the simulation time, the water coordination number increased to two water molecules, which should lead to better contrast and is in accordance with the experimentally derived number of water molecules in the first coordination sphere of lanthanide ions in fish Parvalbumin [11], [12]. In total, the presence of one or two water molecules in the first coordination shell was observed in >95% of the time simulated by MD. During the MD simulations the two functionally important sites of Zarvin, the antibody binding interface and the Gd^3+^-binding site, remained accessible. Taken together, the *in silico* analyses supported that the contrasting and the functional properties of Zarvin are well suited for MRI.

After having obtained evidence for the structural integrity of the expressed Zarvin (Figure S3 and Table S1), we proceeded to test the *in vitro* capability of binding of the Zarvin Z domain to Fc fragments of antibodies. After Recombinantly expressed Zarvin was labelled with Atto-465 at its N-terminus and titrated with the monoclonal anti EGFR-IgG-antibody Cetuximab as a model compound. Changes in fluorescence anisotropy (Figure 1B) revealed a dissociation constant of 470 nM for the complex. To verify the ability of Cetuximab:Zarvin to target cancer cells, binding of this complex to EGFR-expressing cells was demonstrated. The binding of the Cetuximab:Zarvin complex to A431 cancer cells was visualized by confocal microscopy. To this end, a D72C mutant of Zarvin was labeled with Atto-594 and incubated with Cetuximab before the Cetuximab:Zarvin-D72C complex was added to A431 cells. Staining was almost exclusively observed at the cellular membrane (Figure 1C). Fluorescence signals at the inside of the membrane visible at higher magnification could result from internalization of the complexes over time [13]. Thus, we demonstrated that coupling of Zarvin to a therapeutically used antibody resulted in efficient binding to target cells *in vitro* (for the controls see Figure S4).

We then tested the capability of the S55D/E59D alpha-Parvalbumin domain to bind Gd^3+^ with high affinity as a prerequisite for use as a T_1_ contrast agent. Gadolinium(III) and Terbium(III) have a similar ion radius [14] and both are ‘hard’ cations with similar chemical properties according to the HSAB concept. Terbium(III), however, shows detectable luminescence upon binding to a chelating compound. Therefore, the affinity for complex formation of the high affinity EF-site of Zarvin and Tb^3+^ was estimated first in a competition assay using nitrilotriacetic acid (NTA) as a competitor. Using the fitted apparent affinity of NTA:Tb^3+^ and the binding affinity of 5.6 × 10^−12^ M given in the literature for this complex [15], the binding affinity of the EF-site:Tb^3+^ complex was calculated to be in the subpicomolar range (∼3 × 10^−13^ M), whereas the affinity of the CD-site can be regarded as about 5–6 fold lower (Figure S5). The affinities of Gd^3+^ and for comparison of Ca^2+^ were estimated using a competition assay (Figure S5) containing 4 µM Zarvin and 10 µM Tb^3+^. Titration with Gd^3+^ yielded an apparent dissociation constant K_app_ of 25 µM, whereas Ca^2+^ yielded a value of 83 µM leading to binding affinities of 7 × 10^−13^ M for Zarvin:(Gd^3+^)_2_ and 2 × 10^−12^ M for Zarvin:(Ca^2+^)_2_. Thus, Gd^3+^ binds slightly weaker than Tb^3+^ (due to the somewhat larger ionic radius of Gd^3+^), but by a factor of 3.3 stronger than Ca^2+^.The estimated affinity constants confirm that high affinity binding of metal ions is conserved in the two-domain fusion construct.

As Zarvin was designed to function as a targeting T_1_ contrast agent it should have a high relaxivity r_1_, as high r_1_ values correlate with the contrast generated by the agent. The relaxometric properties of Zarvin were measured *in vitro* using whole-body MRI systems at room temperature with field strengths of 1.5 T, 3 T as well as 7 T and. Serial dilutions of Zarvin:(Gd^3+^)_2_ were subjected to an inversion recovery turbo spin echo experiment. In Figure 1D, increasing brightness observed in the wells along each row displays increasing contrast as a function of the Zarvin:(Gd^3+^)_2_ concentration. While at 1.5 T the limit for detecting observable contrast was found at a protein concentration around 0.5 µM, this concentration was shifted to 0.5–1 µM at 3 T and reached values between 1–5 µM at a field strength of 7 T. Longitudinal relaxivities r_1_ of Gd^3+^ ions bound to Zarvin yielded values of 50.6±1.3 s^−1^mM^−1^ for 1.5 T, 24.9±0.5 s^−1^mM^−1^ for 3 T and 8.8±1.5 s^−1^mM^−1^ for 7 T at room temperature, respectively. As relaxivities of conventionally used small molecular contrast agents like DTPA:Gd^3+^ (Magnevist®) and DOTA:Gd^3+^ (Dotarem®) are below 10 s^−1^mM^−1^ irrespective of field strength [16], relaxivity of Zarvin is considerably higher compared to the clinically applied Gd^3+^-chelators, at least at 1.5 and 3 T. This can also be observed in a respective NMRD profile of Zarvin:(Gd^3+^)_2_ recorded at 37°C (Figure S6). Moreover, by decoupling the Parvalbumin domain from the Z domain via the decaglycine linker, r_1_ values of IgG bound Zarvin:(Gd^3+^)_2_ are not reduced at 3 T and 7 T as would be expected for a rigid bound protein species.

From Figure 1D it can be estimated whether the achievable concentrations of Zarvin:(Gd^3+^)_2_ are sufficient to produce observable contrast when bound to A431 cancer cells. This cell line expresses about 1.6–2.6 × 10^6^ EGF receptor molecules per cell [17], [18]. Assuming a cell diameter of 15–25 μm, the concentration of EGF receptors averaged over the volume of a cell is between 0.32 and 2.44 µM. According to this simple model metastases could receive higher contrast than normal tissue at 1.5 or 3 T by using Zarvin(Gd^3+^)_2_ in combination with Cetuximab as a contrast agent instead of commercial available small molecular weight contrast agents. Detection of metastases would then be limited by the resolution of the MRI scanner, which is in the sub-millimetre range for the three field strengths mentioned. Metastases that are large enough to be displayed in the respective MR images, could then be sufficient to produce a detectable contrast towards normal tissues at Zarvin:(Gd^3+^)_2_ protein concentrations of 0.32–2.44 µM inside the metastasis [6].

To test suitability of Zarvin for *in vivo* applications, its stability towards temperature and serum was investigated using fetal calf serum (FCS). Zarvin at a concentration of 2 mg/ml was incubated in 50% FCS at 37°C. Then, aliquots were taken and tested for degradation (Figure S7). Even after 24 h, allowing enough time for MRT examination and subsequent excretion of the contrast agent, there is no visible degradation of the fusion protein. Next, structural integrity of Zarvin at different temperatures was measured employing CD spectroscopy. The CD signal at 225 nm was recorded during heating of the sample (Figure S8). Although the metal ion free apo-form of Zarvin is not stable at body temperature, binding of Gd^3+^ to the EF- and CD-site stabilizes the holo-form of the domain relevant for *in vivo* application. The melting point of Zarvin:(Gd^3+^)_2_ was determined to be >75°C. Zarvin:(Gd^3+^)_2_ refolded completely reversibly, which is an advantage for the shelf life of Zarvin and probably also of its mutants. Kinetic stability as an important predictor for *in vivo* stability of the Zarvin:(Tb^3+^)_2_ complex was investigated by luminescence measurements. In FCS half-lives of about 2.5–3 min were determined for the protein-metal complex. The low half-life is caused by the presence of Ca^2+^ and metal ion binding proteins in the serum. To explore, which of both components is mainly responsible for pulling out of Tb^3+^, serum proteins were separated from the liquid part by ultrafiltration of FCS. Then, dissociation of the Zarvin:(Tb^3+^)_2_ complex was measured in the flow-through as well as in a Tris buffered solution containing the washed serum proteins. In the flow-through the same half-life of Zarvin:(Tb^3+^)_2_ was observed as measured in the presence of total FCS, whereas in the presence of serum proteins the metal ion half-life in the complex was extended to about 90 minutes.

Thus, there are currently two major obstacles for the *in vivo* detection of metastasis by Zarvin that have to be addressed in further studies: Considering the nanomolar affinity constant of the Zarvin:Cetuximab complex and the high concentration of various IgG antibodies in blood serum of humans (denoted IgGx) the complex could undergo rapid dissociation and subsequent Zarvin:IgGx complex formation upon application. Although this could hamper the detection of metastasis, the application of Zarvin could yet be a desired option for the detection of centres of inflammation in patients exhibiting elevated levels of specific antibodies while suffering from autoimmunic diseases or rheumatoid arthritis. A more crucial task to improve Zarvin for *in vivo* applications is to optimize the kinetic stability of the Zarvin:(Gd^3+^)_2_ complex by site directed mutagenesis to cage the metal ion more firmly inside the binding sites.

### Conclusion

We conclude that Zarvin keeps its native conformation at body temperature and is not sensitive to protease degradation in blood serum. Due to its monomeric form and molecular weight Zarvin is likely to be excreted from the system by renal filtration without the need of enzymatic degradation in the blood. Zarvin’s high longitudinal relaxivities combined with its IgG binding feature make it a promising tumour candidate T_1_ contrast agent for targeting tumours and for the highly sensitive detection of metastases in MRI.

## Supporting Information

Figure S1 **Molecular dynamics simulation studies of Zarvin.** Left, boxplot of the secondary structure elements over the MD simulations. Right, histogram of the distances between the Z domain and Parvalbumin.
(TIF)

Figure S2 **Number of water molecules in the first coordination sphere of Ca^2+^ ions of S55D/E59D rat alpha-Parvalbumin in percent.** The plot shows the distribution (black circles and crosses) of one (x-axis) and two (y-axis) water molecules in the first coordination shell of Ca^2+^ during the molecular dynamics simulations as well as the calculated averages (red circle and cross). Points with an exclusive coordination of one water in the first coordination shell (position x = 1 and y = 0)) were separated along the x-axis for better readability.
(TIF)

Figure S3 **Integrity and structure of Zarvin.** A, MALDI mass spectrum of Zarvin yielding a mass of 19156.1 Da (theoretical mass 19156.3 Da for M+H^+^). B, CD spectrum of Zarvin recorded in 20 mM Na_2_PO_4_, pH 7.4 and room temperature. C, Overlay of ^1^H-^15^N-HSQC spectra of Zarvin (green), S55D/E59D rat alpha-Parvalbumin (blue) and the Z domain (red). K58 is the last amino acid of the Z domain and thus shifts within Zarvin due to the Glycine_10_ linker, which now follows after K58. The majority of the resonances of Zarvin align nearly perfectly with those of the single domains. Thus, both domains fold independently and correctly within Zarvin.
(TIF)

Figure S4 **Controls.** Controls of the cell based experiment in which A431 cells were incubated with the complex Zarvin-D72C-Atto-594:Cetuximab (Figure 1C of the main text).
(TIF)

Figure S5 **Affinity measurements of Lanthanide ion binding.** Left, titration of 3 µM TbCl_3_ and 8 µM Zarvin with NTA. Luminescence of Terbium (III) was recorded. The curve was normalized and inverted prior to fitting. It was known from an active site titration that binding of Tb^3+^ to the EF-site (5–6 × higher Ca^2+^ affinity than the CD-site^4^) contributes to a larger amount to the overall luminescence measured (approximately 5×). This effect produces a quasi-cooperative behavior of the luminescence signal upon titration with NTA, forming a sigmoidal curve. The global IC_50_ value of the curve does not vary to a large extent when either estimated or fitted as a K_app_ using a Hill equation due to the sigmoidal shape of the curve. K_app_ stands for the apparent K_D_ of the NTA:Tb^3+^ complex in the presence of Zarvin. As the luminescence contribution of Tb^3+^ in the EF-site dominates the global IC_50_/K_app_ value, an approximately 5–6× lower affinity can be assumed for the CD-site, analogous to the binding behavior of Ca^2+^. Right, competition titration of 4 µM Zarvin and 10 µM TbCl_3_ with GdCl_3_ and CaCl_2_, respectively. Luminescence of Terbium(III) was recorded. K_app_ stands for the apparent K_D_ of the Zarvin:Gd^3+^ or Zarvin:Ca^2+^ complexes in the presence of Tb^3+^.
(TIF)

Figure S6 **NMRD profile of Zarvin:(Gd^3+^)_2_.** The NMRD profile confirms the high efficiency of Zarvin:(Gd^3+^)_2_ measured at clinically used MRI devices in terms of the relaxivity r_1_. The relaxivity of Zarvin:(Gd^3+^)_2_ at 1.5 Tesla and 37°C (between the last and last but one point in the profile referring to 60 and 70 MHz, respectively, and considering 42.58 MHz per Tesla) is a factor of 5–10 above relaxivities of clinically used small molecular weight contrast agents.
(TIF)

Figure S7 **Stability of Zarvin in 50% FCS and 37°C over time.** Aliquots were taken after distinct times (displayed in minutes) and investigated on degradation of Zarvin employing a Schägger-Jagov gel. The lanes denoted ‘50% FCS’ serve as a control and do not contain Zarvin. The lanes denoted “Z/P” serve as a control as well and contain Zarvin only. M: Mark 12.
(TIF)

Figure S8 **Melting curves of Zarvin and single domains.** CD spectra at 225 nm were recorded while temperature was raised from 15 to 95°C. The thermal stability of Zarvin (blue), Zarvin:(Gd^3+^)_2_ (black) and the single domains S55D/E59D alpha-Parvalbumin (green) and Z domain (red) could be characterized by extracting melting points from the first derivative of the melting curves.
(TIF)

Table S1 **Data collection and refinement statistics.** Calculated and measured secondary structure elements from DSSP and CD spectroscopy, respectively.
(DOCX)
