# Supplementary figures and images for: Design of a Modular Protein-Based MRI Contrast Agent for Targeted Application
Source: PLoS One. 2013 Jun 6;8(6):e65346. doi: 10.1371/journal.pone.0065346 (PMC3675113; doi:10.1371/journal.pone.0065346)

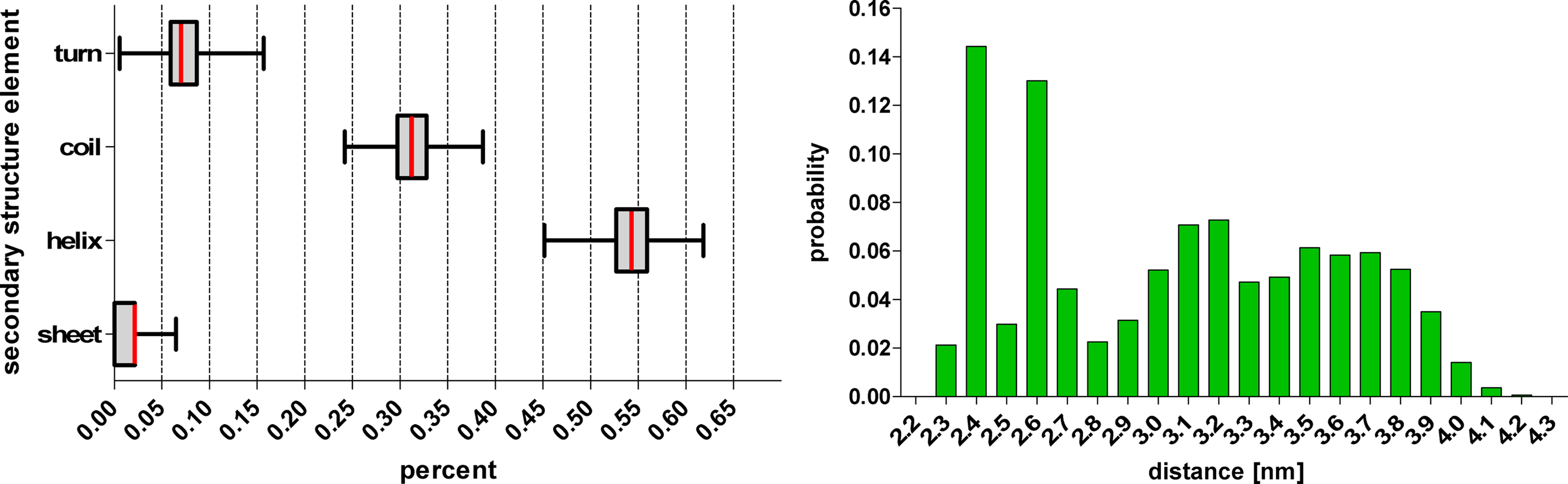

Supplement: Figure S1 — Molecular dynamics simulation studies of Zarvin. Left, boxplot of the secondary structure elements over the MD simulations. Right, histogram of the distances between the Z domain and Parvalbumin. (TIF) [file pone.0065346.s001.tif]

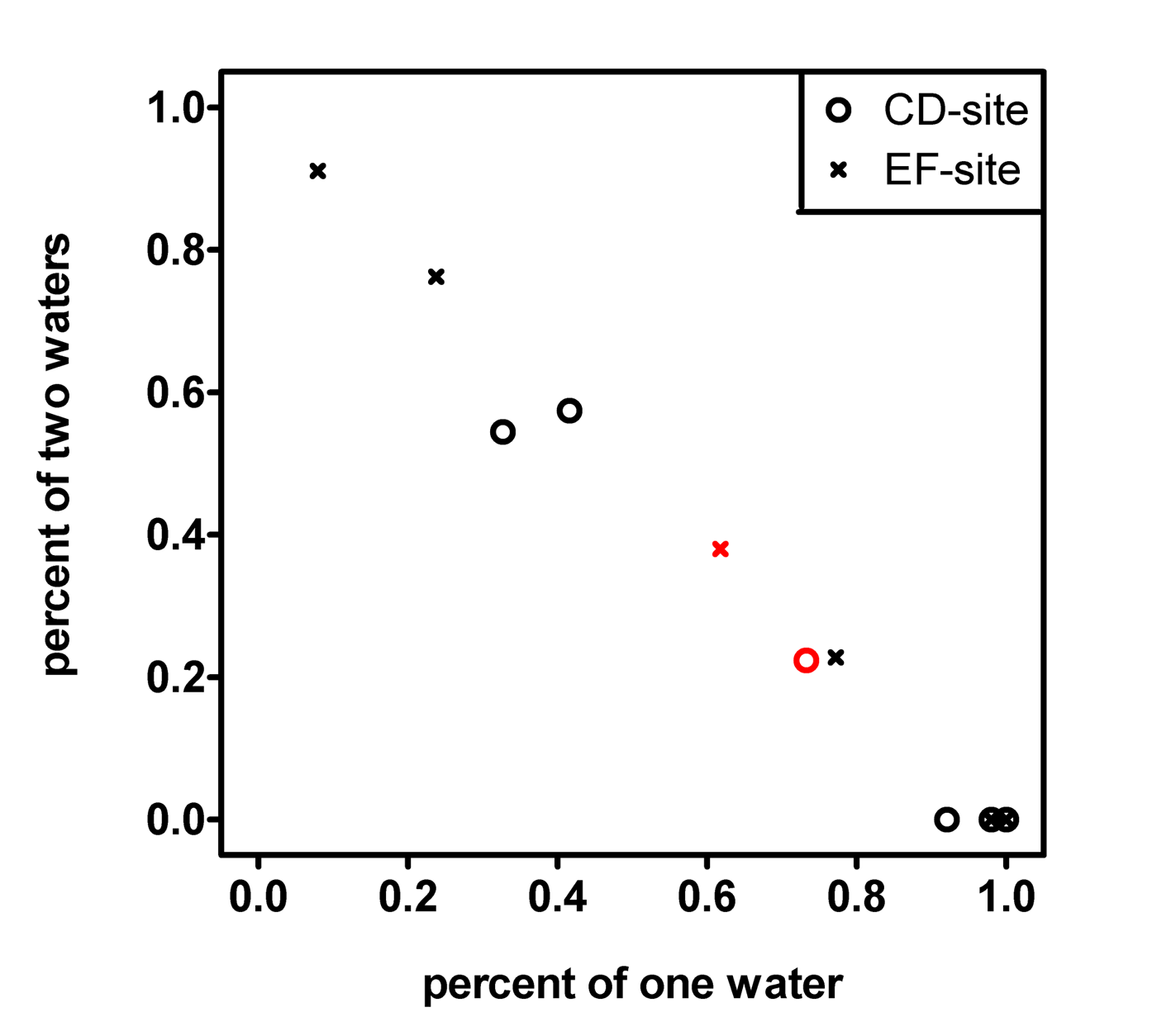

Supplement: Figure S2 — Number of water molecules in the first coordination sphere of Ca2+ ions of S55D/E59D rat alpha-Parvalbumin in percent. The plot shows the distribution (black circles and crosses) of one (x-axis) and two (y-axis) water molecules in the first coordination shell of Ca2+ during the molecular dynamics simulations as well as the calculated averages (red circle and cross). Points with an exclusive coordination of one water in the first coordination shell (position x = 1 and y = 0)) were separated along the x-axis for better readability. (TIF) [file pone.0065346.s002.tif]

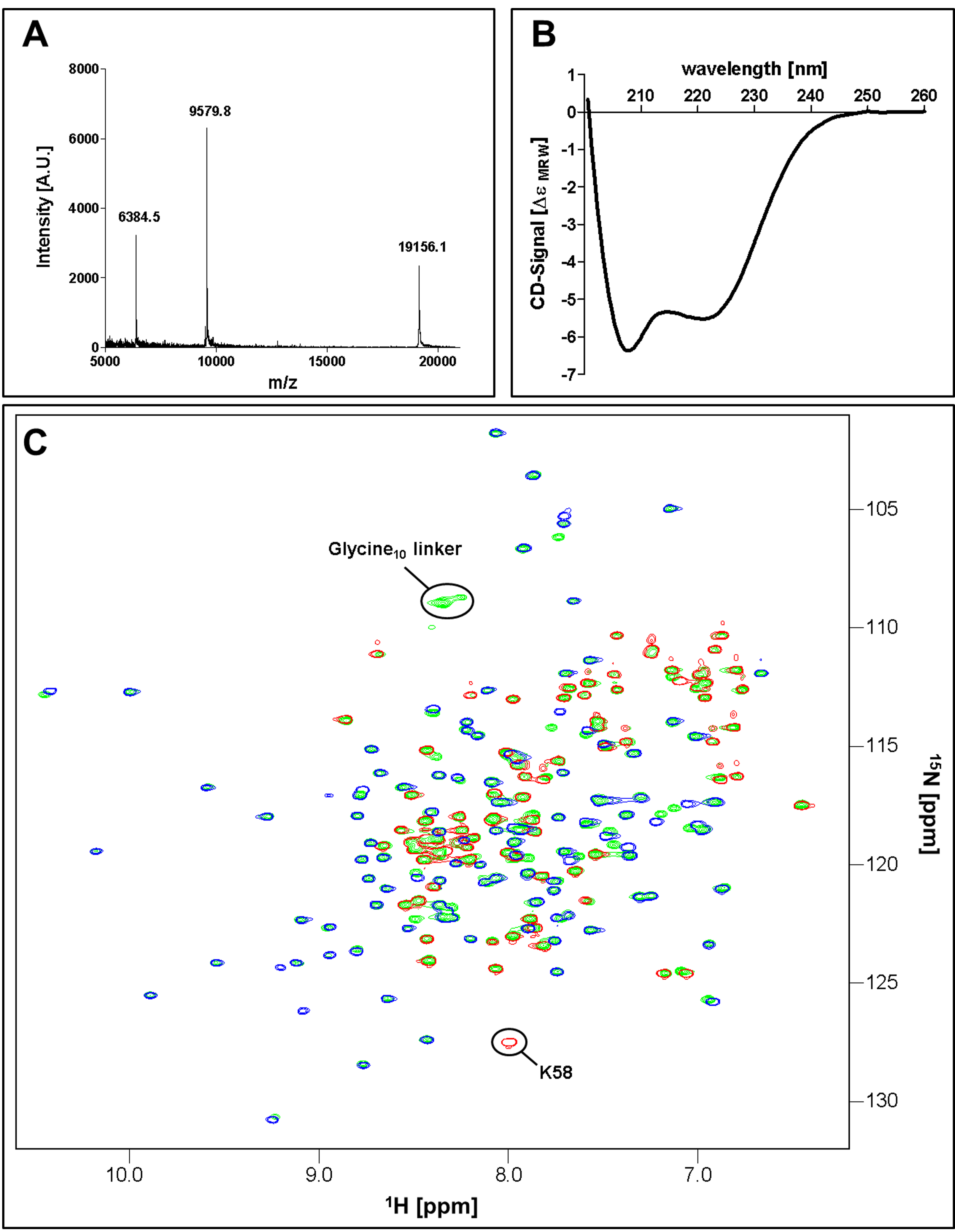

Supplement: Figure S3 — Integrity and structure of Zarvin. A, MALDI mass spectrum of Zarvin yielding a mass of 19156.1 Da (theoretical mass 19156.3 Da for M+H+). B, CD spectrum of Zarvin recorded in 20 mM Na2PO4, pH 7.4 and room temperature. C, Overlay of 1H-15N-HSQC spectra of Zarvin (green), S55D/E59D rat alpha-Parvalbumin (blue) and the Z domain (red). K58 is the last amino acid of the Z domain and thus shifts within Zarvin due to the Glycine10 linker, which now follows after K58. The majority of the resonances of Zarvin align nearly perfectly with those of the single domains. Thus, both domains fold independently and correctly within Zarvin. (TIF) [file pone.0065346.s003.tif]

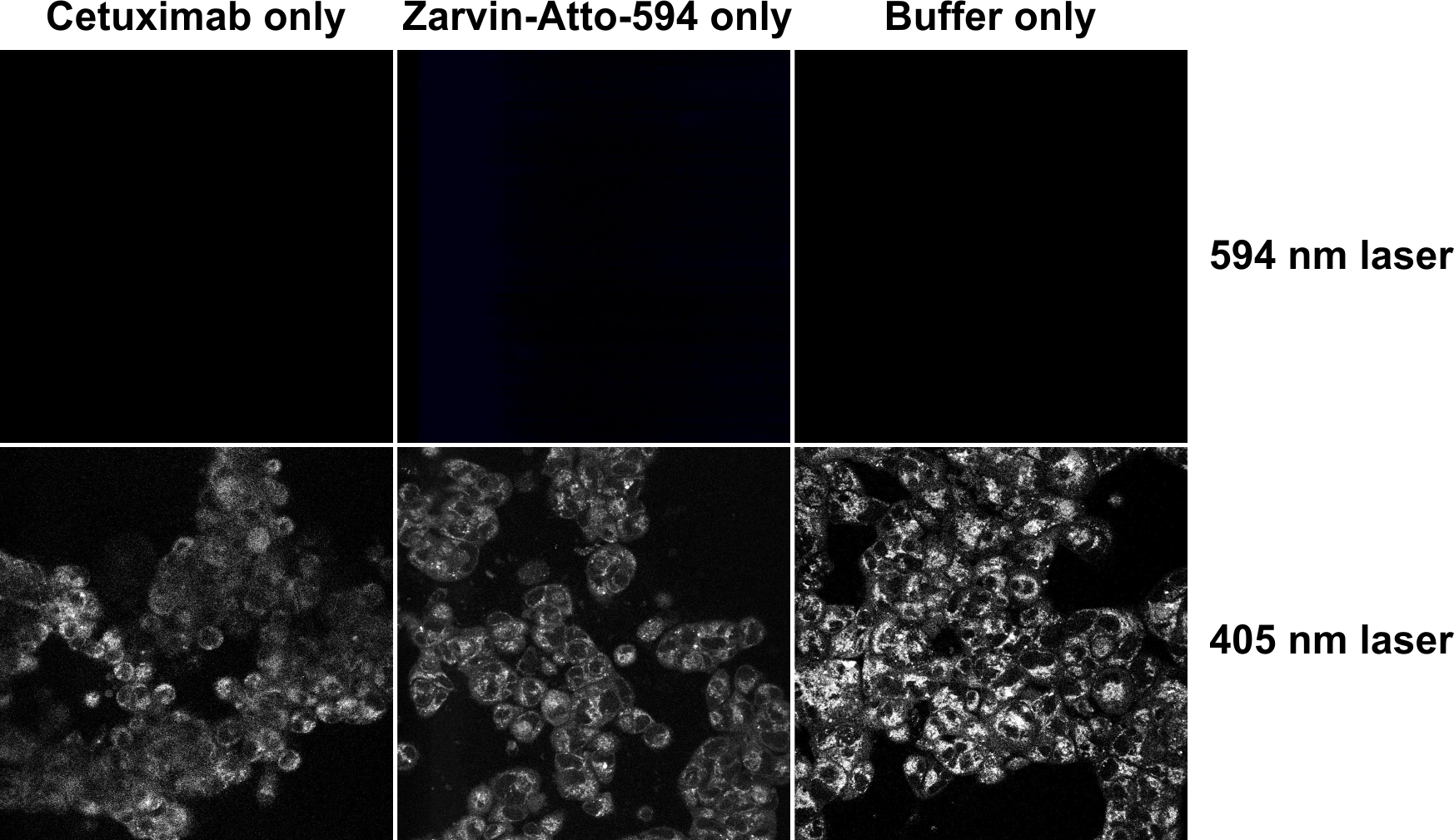

Supplement: Figure S4 — Controls. Controls of the cell based experiment in which A431 cells were incubated with the complex Zarvin-D72C-Atto-594:Cetuximab (Figure 1C of the main text). (TIF) [file pone.0065346.s004.tif]

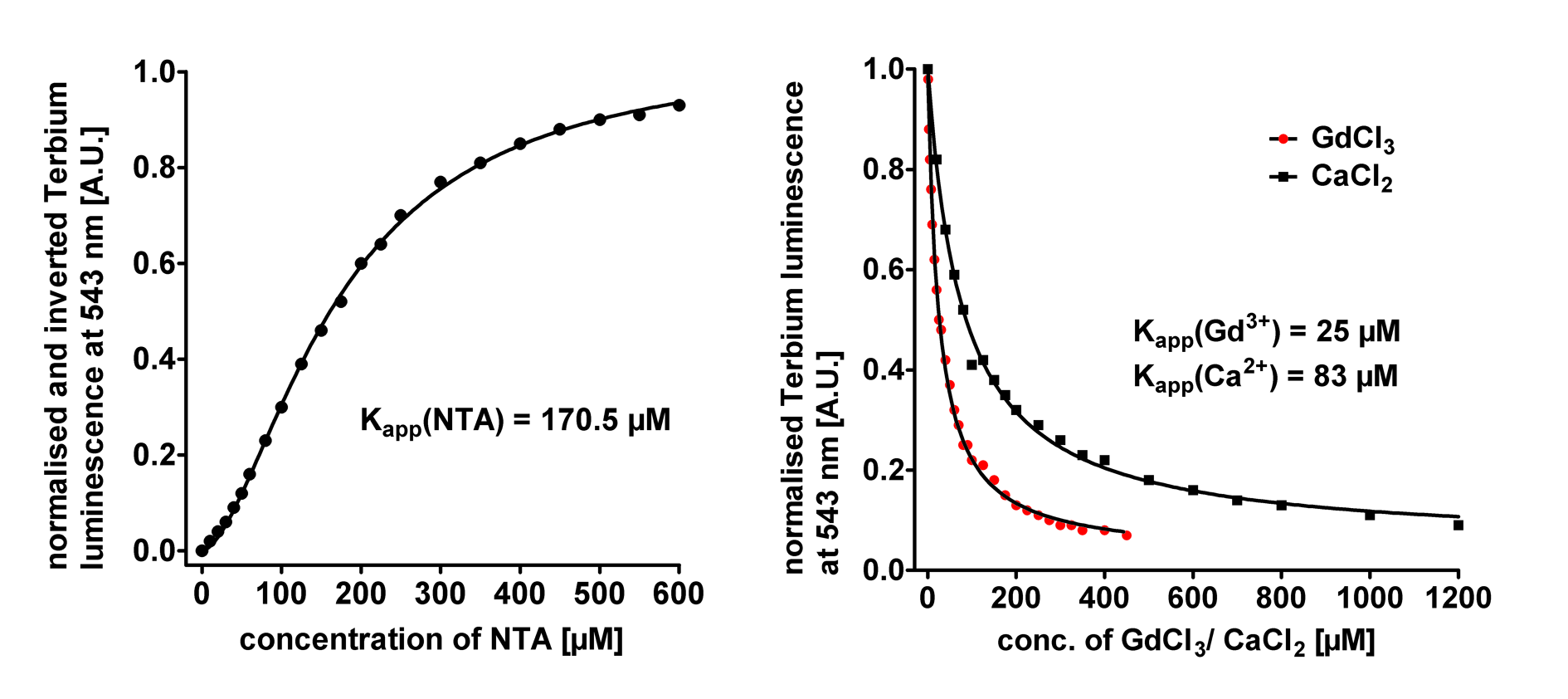

Supplement: Figure S5 — Affinity measurements of Lanthanide ion binding. Left, titration of 3 µM TbCl3 and 8 µM Zarvin with NTA. Luminescence of Terbium (III) was recorded. The curve was normalized and inverted prior to fitting. It was known from an active site titration that binding of Tb3+ to the EF-site (5–6 × higher Ca2+ affinity than the CD-site4) contributes to a larger amount to the overall luminescence measured (approximately 5×). This effect produces a quasi-cooperative behavior of the luminescence signal upon titration with NTA, forming a sigmoidal curve. The global IC50 value of the curve does not vary to a large extent when either estimated or fitted as a Kapp using a Hill equation due to the sigmoidal shape of the curve. Kapp stands for the apparent KD of the NTA:Tb3+ complex in the presence of Zarvin. As the luminescence contribution of Tb3+ in the EF-site dominates the global IC50/Kapp value, an approximately 5–6× lower affinity can be assumed for the CD-site, analogous to the binding behavior of Ca2+. Right, competition titration of 4 µM Zarvin and 10 µM TbCl3 with GdCl3 and CaCl2, respectively. Luminescence of Terbium(III) was recorded. Kapp stands for the apparent KD of the Zarvin:Gd3+ or Zarvin:Ca2+ complexes in the presence of Tb3+. (TIF) [file pone.0065346.s005.tif]

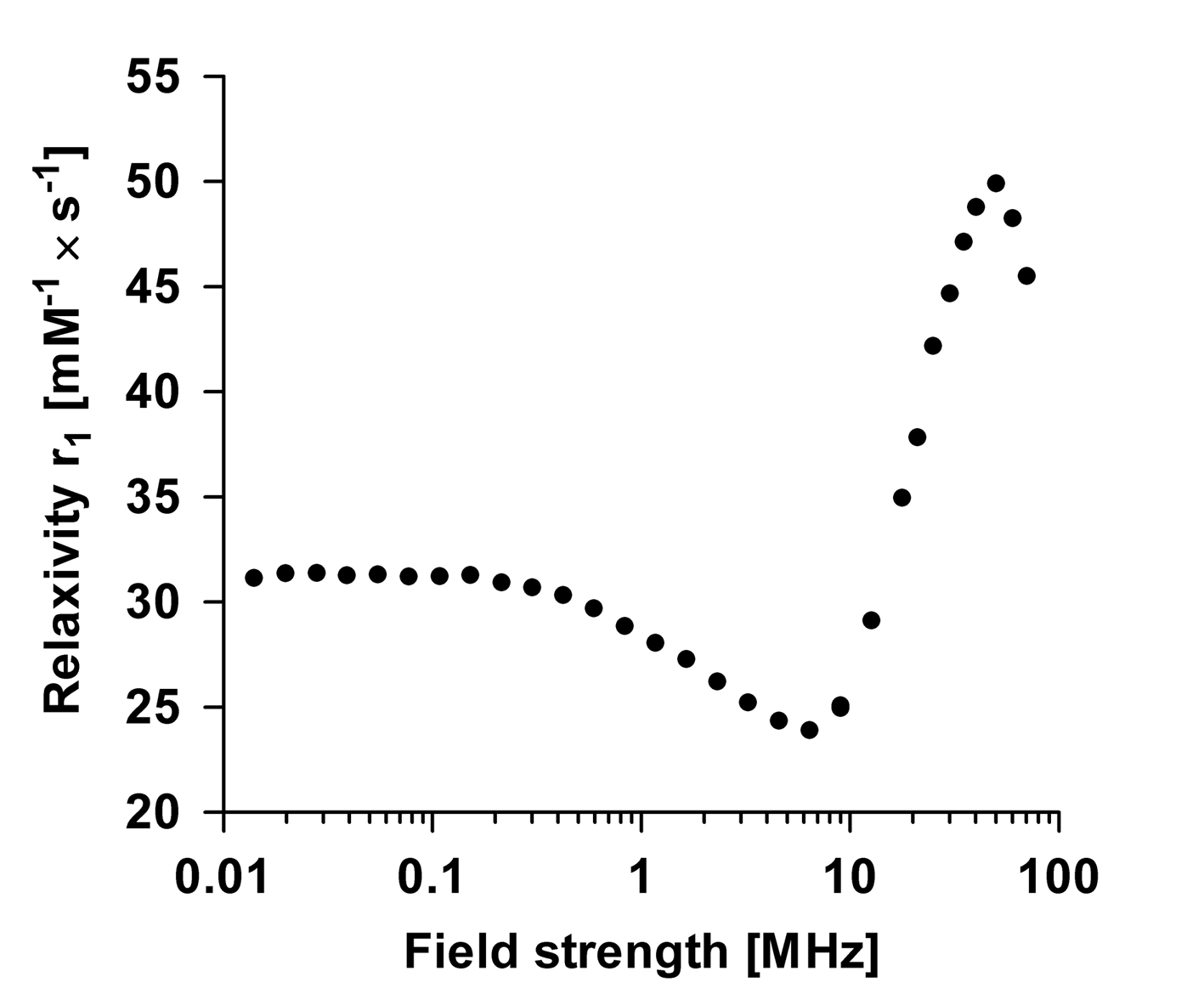

Supplement: Figure S6 — NMRD profile of Zarvin:(Gd3+)2. The NMRD profile confirms the high efficiency of Zarvin:(Gd3+)2 measured at clinically used MRI devices in terms of the relaxivity r1. The relaxivity of Zarvin:(Gd3+)2 at 1.5 Tesla and 37°C (between the last and last but one point in the profile referring to 60 and 70 MHz, respectively, and considering 42.58 MHz per Tesla) is a factor of 5–10 above relaxivities of clinically used small molecular weight contrast agents. (TIF) [file pone.0065346.s006.tif]

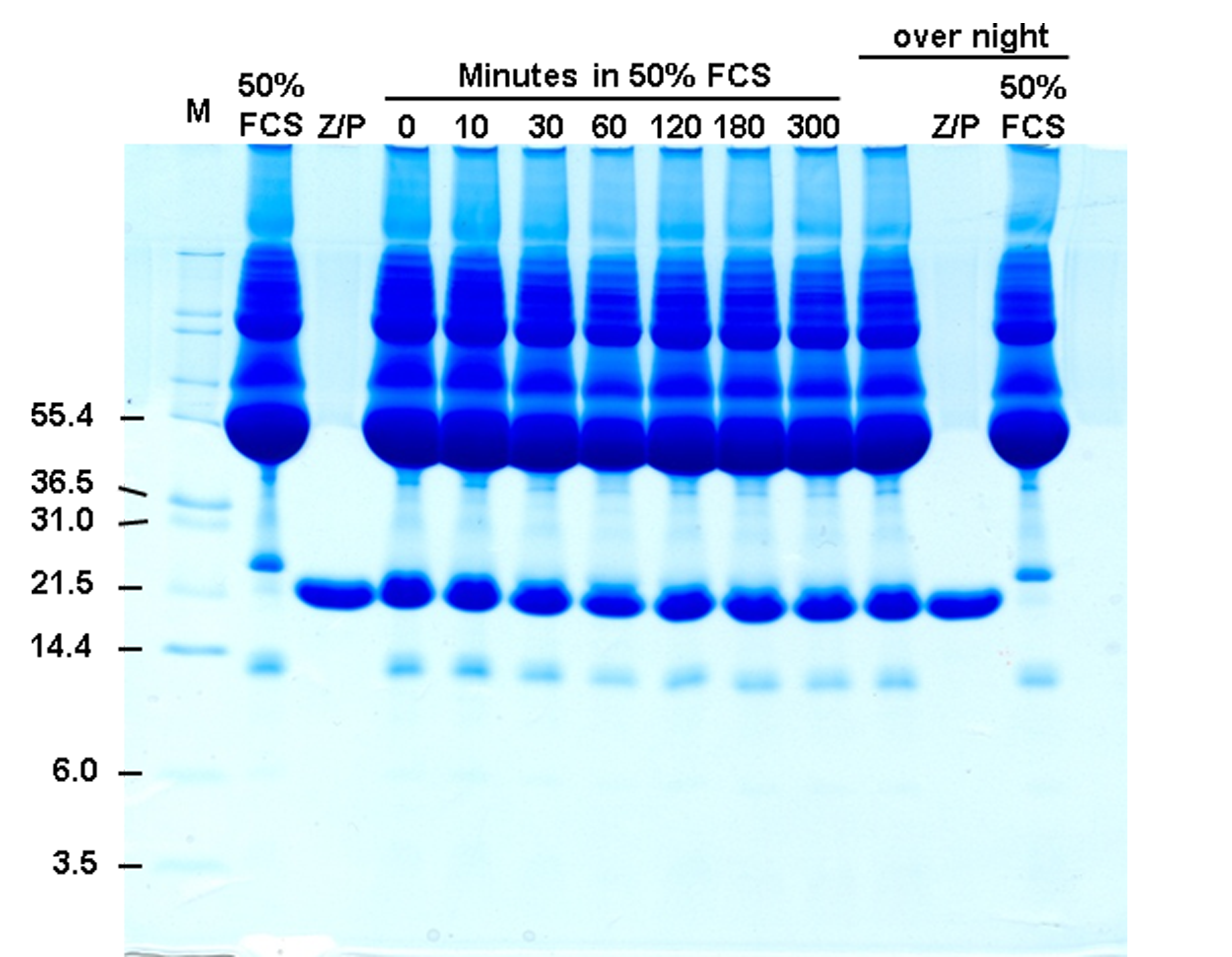

Supplement: Figure S7 — Stability of Zarvin in 50% FCS and 37°C over time. Aliquots were taken after distinct times (displayed in minutes) and investigated on degradation of Zarvin employing a Schägger-Jagov gel. The lanes denoted ‘50% FCS’ serve as a control and do not contain Zarvin. The lanes denoted “Z/P” serve as a control as well and contain Zarvin only. M: Mark 12. (TIF) [file pone.0065346.s007.tif]

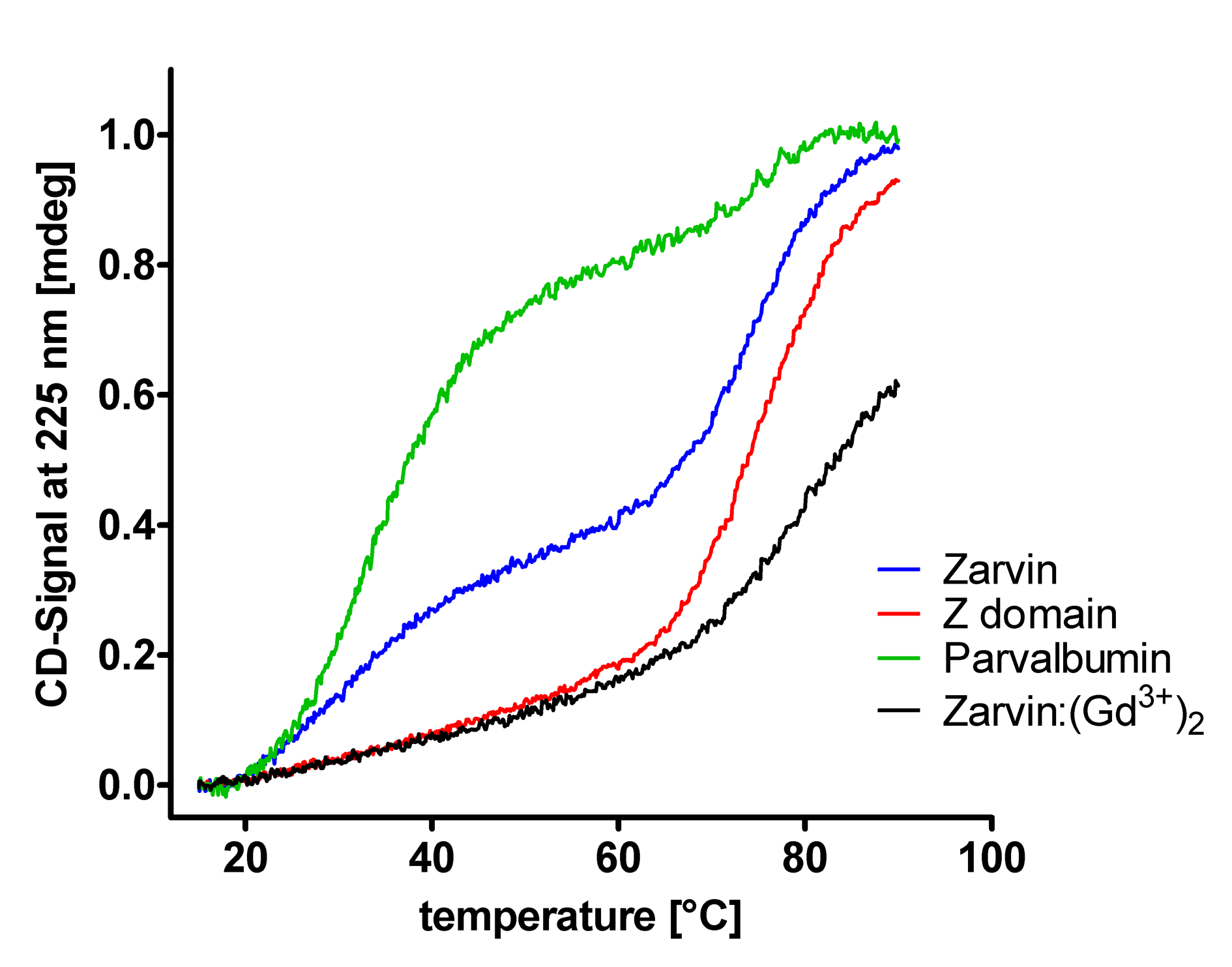

Supplement: Figure S8 — Melting curves of Zarvin and single domains. CD spectra at 225 nm were recorded while temperature was raised from 15 to 95°C. The thermal stability of Zarvin (blue), Zarvin:(Gd3+)2 (black) and the single domains S55D/E59D alpha-Parvalbumin (green) and Z domain (red) could be characterized by extracting melting points from the first derivative of the melting curves. (TIF) [file pone.0065346.s008.tif]
