# Supplementary material for: Design of a Modular Protein-Based MRI Contrast Agent for Targeted Application
Source: PLoS One. 2013 Jun 6;8(6):e65346. doi: 10.1371/journal.pone.0065346 (PMC3675113; doi:10.1371/journal.pone.0065346)
Supplement: Table S1 — Data collection and refinement statistics. Calculated and measured secondary structure elements from DSSP and CD spectroscopy, respectively. (DOCX) [file pone.0065346.s009.docx]

|  | helix | sheet | turn | coil (unordered) |
| --- | --- | --- | --- | --- |
| DSSP | 64.3% | 2.4% | 9.6% | 25.8% |
| CD spectroscopy | 61.8 – 64.7% | 1.4 – 3.7% | 7.1 – 12.1% | 23.7 – 24.7% |
